# In vivo imaging of mGlu5 receptor expression in humans with Fragile X Syndrome towards development of a potential biomarker

**DOI:** 10.1038/s41598-021-94967-y

**Published:** 2021-08-05

**Authors:** Maria Mody, Yoann Petibon, Paul Han, Darshini Kuruppu, Chao Ma, Daniel Yokell, Ramesh Neelamegam, Marc D. Normandin, Georges El Fakhri, Anna-Liisa Brownell

**Affiliations:** 1 Athinoula A Martinos Center for Biomedical Imaging, Massachusetts General Hospital and Harvard Medical School, Boston, MA 02129 USA; 2 Gordon Center for Medical Imaging, Massachusetts General Hospital and Harvard Medical School, Boston, MA 02129 USA; 3 Department of Surgery, Massachusetts General Hospital and Harvard Medical School, Boston, MA 02129 USA; 4 Department of Radiology, University of Texas Health Science at San Antonio, San Antonio, TX 78229 USA

**Keywords:** Neuroscience, Biomarkers

## Abstract

Fragile X Syndrome (FXS) is a neurodevelopmental disorder caused by silencing of the Fragile X Mental Retardation (*FMR1*) gene. The resulting loss of Fragile X Mental Retardation Protein (FMRP) leads to excessive glutamate signaling via metabotropic glutamate subtype 5 receptors (mGluR5) which has been implicated in the pathogenesis of the disorder. In the present study we used the radioligand 3-[18F]fluoro-5-(2-pyridinylethynyl)benzonitrile ([^18^F]FPEB) in simultaneous PET-MR imaging of males with FXS and age- and gender-matched controls to assess the availability of mGlu5 receptors in relevant brain areas. Patients with FXS showed lower [^18^F]FPEB binding potential (p <  0.01), reflecting reduced mGluR5 availability, than the healthy controls throughout the brain, with significant group differences in insula, anterior cingulate, parahippocampal, inferior temporal and olfactory cortices, regions associated with deficits in inhibition, memory, and visuospatial processes characteristic of the disorder. The results are among the first to provide in vivo evidence of decreased availability of mGluR5 in the brain in individuals with FXS than in healthy controls. The consistent results across the subjects, despite the tremendous challenges with neuroimaging this population, highlight the robustness of the protocol and support for its use in drug occupancy studies; extending our radiotracer development and application efforts from mice to humans.

## Introduction

Fragile X Syndrome (FXS) is a leading cause of inherited intellectual disability^1^ and the most common, known single gene cause of autism spectrum disorder^2,3^. Successful treatment of FXS has the potential to positively impact the larger field of neurodevelopmental disabilities. A number of studies have implicated metabotropic glutamate subtype 5 receptors (mGluR5) in FXS^4^; the use of mGluR5 antagonists in Fmr1 Knockout (KO) mouse models has demonstrated a variety of benefits including reduced seizures, anxiety, and behavioral issues^5,6^. The Fmr1 KO mouse model has shown that the absence of FMRP leads to increased protein synthesis at the synapse and excessive mGluR5 glutamatergic signaling^7^, building on the work of Weiler and colleagues who first identified the connection between FMRP and mGluR5 pathways^8^. However, despite these promising preclinical findings, clinical trials of mGluR5-targeted drugs in FXS have met with limited success. It is unclear as to whether these trials were conducted with optimal outcome endpoints or in the most appropriate age group^9–11^.

The severity of FXS phenotype is known to be correlated with the magnitude of the FMRP deficit^12^. The *FMR1* gene responsible for FXS is located on the X chromosome, thereby affecting males with FXS more severely than their female counterpart who have one unaffected X chromosome to fall back on^13^. We focused on measuring the availability and distribution of mGluR5 in males with FXS and age- and gender-matched healthy controls to better understand the role of mGluR5 expression in the pathophysiology of FXS in humans. Given the heterogeneity found in neurodevelopmental disorders like FXS, stringent methodological standards were used to obtain reliable delineation and quantification of mGluR5 occupancy differences between healthy and disorder groups, critical for biomarker research.

In the present study, we examined PET data from five adult males with FXS (> 200 CGG repeats, full mutation, 26–48 years of age; mean: 34.8 years ± 7.8) and seven age-matched males with normal development (22–43 years of age; mean 31.4 years ± 7.5) from whom PET and MRI data was successfully obtained (see “Methods” for details). Participants with FXS were recruited from the hospital database, local clinics and Fragile X organizations; typical controls (TC) were drawn from the local community. All subjects completed a high resolution MRI and 90-min dynamic PET examination on an integrated whole-body PET-MR (Siemens Biograph mMR) following an intravenous bolus injection of 5 mCi of [^18^F]FPEB, a safe and reliable radioligand for quantifying regional brain concentrations of mGluR5^14^. Based on *Fmr1* mouse studies of the mGluR theory and the established affinity and specificity of [^18^F]FPEB to mGluR5, we hypothesized that there would be a significant difference in regional brain concentrations of mGluR5 between the two groups of subjects, validating the use of [^18^F]FPEB in clinical trials as a tool to confirm target engagement of novel drug agents for mGluR5s in FXS and to monitor dose response.

In a recent study, Brašić and colleagues^17^ used the same [^18^F]FPEB tracer in males with FXS, slightly younger than those in the present study, though incorporating different acquisition protocols and data analysis methods for PET across collaborating sites; they found mGluR5 density was significantly reduced in multiple brain regions including the cingulate, cortex, thalamus and striatum, compared to age-matched males with typical development, thereby supporting the use of [^18^F]FPEB to measure drug occupancy in clinical trials for mGLuR5 in FXS.

## Results

**Figure 1:**
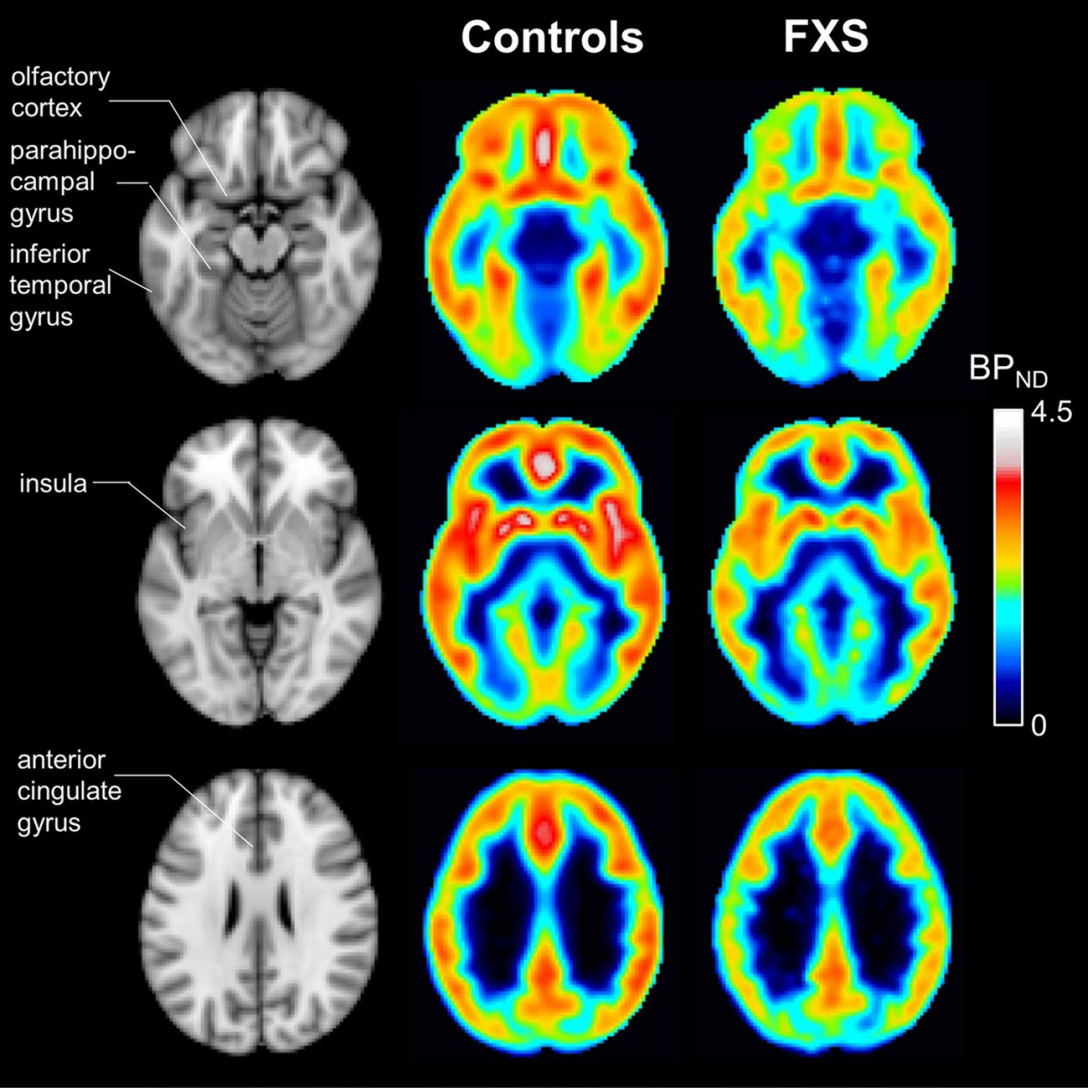
Group-average images of [^18^F]FPEB BP_ND_ in control subjects and subjects with FXS, and corresponding MR template images. Regions exhibiting significant differences in BP_ND_ between control subjects and subjects with FXS (olfactory cortex, parahippocampal gyrus, inferior temporal gyrus, insula, and anterior cingulate gyrus) are indicated in the MR images.

Regional and voxel-wise estimates of [^18^F]FPEB non-displaceable binding potential (BP_ND_), a measure of mGluR5 availability, were obtained for each subject by multi-linear reference tissue analysis of dynamic PET data^15,16^. We found regional brain uptake was consistent with known mGlu5 receptors based on previous studies^1,17^. A global reduction of [^18^F]FPEB BP_ND_ was observed in males with FXS compared to control subjects (Fig. 1).

**Figure 2:**
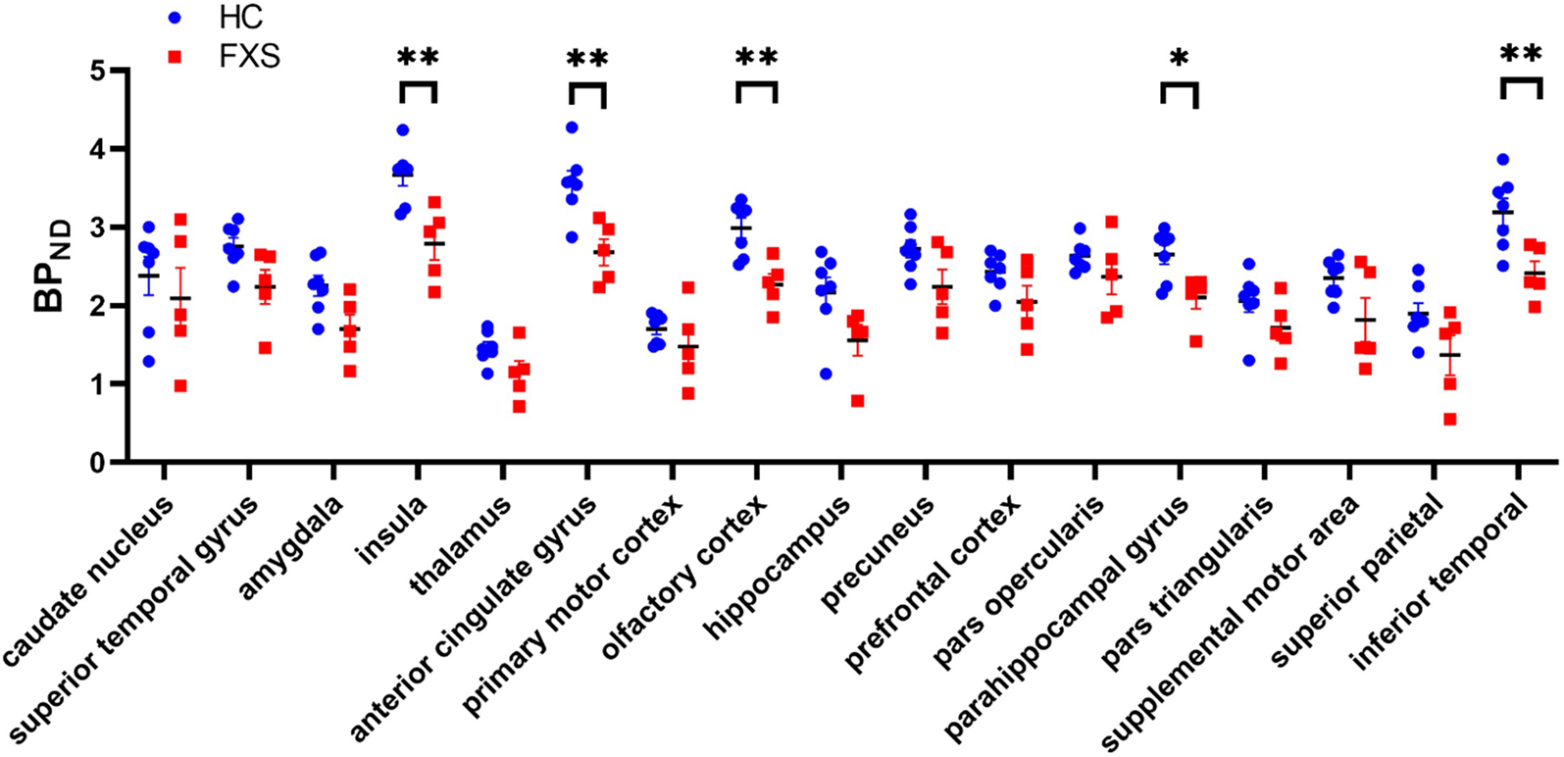
Regional [^18^F]FPEB BP_ND_ in control subjects (blue circle) and subjects with FXS (red square). Error bars indicate standard error of the means. *p < 0.05, **p < 0.01 (corrected for multiple comparisons).

Region of interest analyses confirmed BP_ND_ differences between FXS and control groups (Fig. 2). [^18^F]FPEB BP_ND_ were significantly lower in subjects with FXS compared to controls in the anterior cingulate gyrus (2.67 ± 0.38 vs. 3.56 ± 0.41; *p* = 0.004), insula (2.79 ± 0.47 vs. 3.67 ± 0.36; *p* = 0.009), inferior temporal gyrus (2.41 ± 0.34 vs. 3.19 ± 0.47; *p* = 0.008), parahippocampal gyrus (2.10 ± 0.32 vs. 2.65 ± 0.32; *p* = 0.018) and olfactory cortex (2.27 ± 0.30 vs. 2.98 ± 0.34; *p* = 0.004) (two-tailed unpaired t-tests, corrected for multiple comparisons using FDR^18^ (*q* = 0.05). BP_ND_ was also significantly lower for subjects with FXS subjects in the amygdala (1.70 ± 0.41 vs. 2.25 ± 0.34; p = 0.04), although it did not survive correction for multiple brain region testing. The findings correspond to those in the neuroimaging literature on FXS^19,20^.

## Discussion

Among the core symptoms of FXS is a deficit in visuospatial learning and memory mediated by hippocampus and parahippocampal and inferior temporal regions^21,22^. Our findings of significantly lower [^18^F]FPEB binding potential (BP_ND_) in these areas provide support for the role of mGluR5 in the neurobiology of FXS. Anxiety is frequently reported in this population, which may explain the significant group differences we observed in the insula and olfactory cortex^19,23^. Evidence in support of these findings also comes from preclinical studies of the Fmr1 KO mouse using the Morris water maze task and the mirrored chamber test^24–26^. Whereas only one of our participants with FXS was on prescription medication for anxiety, a high rate of anxiety in FXS, especially social anxiety, is reported in the literature regardless of gender^12,27,28^. The amygdala group differences we observed did not hold up after multiple comparison, but would likely have survived correction in a larger sample. Thus, the finding clearly suggests a need for larger studies, perhaps a narrower age range, and outcome measures to fully capture the profile of the disorder in males and females. Executive functions have also been consistently documented as impaired in FXS^29^, implicating the prefrontal cortex. While this area did not show a signal in the present study, it deserves to be examined, especially the gyrus rectus which has been shown to have lower levels of FMRP in individuals with FXS than in controls^13^. Taken together, these approaches along with the genetic etiology of FXS will help advance our understanding of gene-brain-behavior relationships in FXS^30^ and related neurodevelopmental conditions like autism spectrum disorder^31^.

The role of mGluR5 has been extensively studied in mouse models of FXS. However, findings related to mGluR5 expression in animals and autopsies of humans with FXS have been inconsistent. And clinical trials targeting mGluR5 in FXS have met with repeated failure, pointing to a need for better designed studies and reliable biomarkers in individuals with FXS^9,32^.

FXS is currently treated symptomatically using behavioral, educational and psychopharmacological strategies^33^ often with unsatisfying results^34,35^. A targeted treatment is lacking.The results of the present study are important as they pave the way for successful human trials by providing a potential biomarker of the impaired mGluR5 mechanism for use in developing targeted drug therapies in FXS. Specifically, using the radioligand [^18^F]FPEB, which has been shown to reliably bind to mGlu5 receptors, we found the binding potential to mGlu5 receptors to be lower throughout the brain in participants with FXS compared with typical controls. The reduced BP appears to reflect reduced density of unoccupied mGlu5 receptors, potentially a downstream consequence of excessive mGluR5 signaling caused by the lack of FMRP protein in individuals with FXS. However, given the lack of consistency of mGlu5 receptor expression findings across human and mouse models, more studies are needed to understand the functional relationship between these variables. Importantly, this difference in BP_ND_ between the subjects with FXS and control group was significant in areas consistent with known distribution of mGluR5, and which have been functionally implicated in FXS symptomatology. Our results of overall reduced mGlu5 receptor expression in relevant regions of the brain in individuals with FXS replicate recent findings^17^ and advance ongoing efforts for a much-needed measure of mGluR5 target engagement for drugs and treatment of symptoms in clinical trials of FXS and related disorders^36,37^.

The participants with FXS in the study had the full Fmr1 mutation with CGG repeats greater than 200. However, some individuals with FXS may present with mosaicism (size or methylation mosaics), which can affect FMRP levels and hence the severity of the condition^38^. There has been significant progress in understanding the physiological role of mGluR5, but treatment with selective mGluR5 antagonists (e.g., MPEP, CTEP, fenobam) have met with limited success in FXS^6,39,40^ despite evidence for use of this approach^41^. Factors like age are known to influence treatment efficacy as was seen when MPEP was administered to 2-week- vs. 6-week-old Fmr1 KO mice; the immature morphological phenotype of pyramidal neurons in the somatosensory cortex were rescued in the younger and less so in the older KO mice.

Insofar as the precise modulation of synaptic transmission either through the use of selective mGluR5 inhibitors to reduce synaptic excitability or through activation of presynaptic GABA_B_ receptors is critical for drug efficacy, [^18^F]FPEB binding potential, an index of mGluR5 availability, may prove to be an invaluable biomarker for optimizing the outcomes of drug trials and improving the lives of individuals with FXS^42^. None of the participants with FXS in the study had an autism diagnosis. However, given the high co-morbidity of FXS and ASD^43^, the use of [^18^F]FPEB to examine differences in mGluR5 binding potential between these disorder groups could significantly contribute to our understanding of the differences in underlying pathophysiology of the disorders; towards development of mechanism-based novel therapeutics in neurodevelopmental disorders. In one such study, Brašić and colleagues^37^, using [^18^F]FPEB, replicated their earlier findings with FXS; but found that compared to subjects with typical development (TD), individuals with autism spectrum disorder (ASD) had higher mGluR5 expression in cortical areas and no difference in subcortical regions. The participants with ASD were younger than the controls. The PET data were acquired and analyzed at collaborating sites using different scanners, scan protocols and analysis methods, as in the original study. Despite potential methodological confounds, these preliminary findings hint at exciting new opportunities for the development of drugs targeting the observed differences in the underlying pathophysiology of the two disorders.

The failure of a clinical trial may not necessarily reflect a failure to understand the underlying mechanism but rather may reveal treatment parameters to be optimized beyond target identification. Future studies could draw on the protocol and findings from the current study to systematically interrogate larger samples with FXS to address questions of disorder severity, age and cognitive abilities as they relate to mGluR5 availability and its regional distribution in the brain. Zhao and colleagues^44^ showed that by taking Nutlin-3, an experimental cancer drug which serves as an inhibitor, mice with FXS lacking the FMRP protein regained their ability to remember what they had seen or smelled in their first study session; this apparent reversal of a memory impairment appeared to specifically relate to reactivation of FMRP affecting neural stem cells and new neurons that they form in the hippocampus. In a recent study, Berry-Kravis and colleagues^45^ found that a phosphodiesterase-4D (PRE4D) allosteric modulator helped improve cognitive functions and behavioral outcomes in patients with FXS. Taken together with the results from the present study, the field of FXS appears poised for major breakthroughs in treatment using inhibitors targeting increased mGluR5 signaling or cyclic AMP (cAMP) production; offering promising new directions for future pharmacological research, as well as approaches to the challenges associated with scanning this population.

In conclusion, the current study supports a role for reduced mGluR5 expression in the underlying pathophysiology of FXS in humans; it holds promise as a potential biomarker for use in clinical trials of FXS that could prove critical in improving pharmacological interventions. Importantly, it builds on our research over the years, from the early development of [^18^F]FPEB as a PET tracer^46^, to its application in the mouse model^47^, and in humans^48^ and most recently, to humans with FXS evident in the present study.

## Methods

### Study subjects

The study was approved by the Institutional Review Board at Massachusetts General Hospital; and performed in keeping with the Human Subjects Research Committee guidelines, and in accordance with the Declaration of Helsinki. We enrolled eight males with FXS and eight age- and gender-matched Controls. Of the eight participants recruited in each group, three with FXS were unable to complete the PET-MR protocol and had to be excluded, as also the data from one Control participant due to technical issues. Subjects with FXS were recruited through the hospital’s patient database, local clinics, and Fragile X organizations. Those with confirmed FMR1 full mutation (> 200 CGG repeats, based on medical records) at screening were eligible to participate. They ranged in age from 26 to 48 years (mean: 34.8 years ± 7.8), had no comorbid diagnosis and were not on any antipsychotic medications. Two of the participants with FXS were on medication (viz., Losartan, Famotidine) for blood pressure and indigestion, and a third on Ritalin (belonging to the stimulant class of drugs) for ADHD symptoms, and Clonazepam (belonging to benzodiazepines class of drugs), as needed, when anxious. While the caregivers reported a tendency for participants to be anxious in new settings and with strangers, only one of the participants with FXS was on prescription medication for anxiety on an as-needed basis. Control participants were recruited from the local community. They had no history of neurological or psychological problems and were not on any prescription medication; they had completed at least two years of college, and were between 22 and 43 years (mean: 31.4 years ± 7.5). The two groups did not differ in age (*p* = 0.416). All participants provided informed consent and/or assent prior to the experiment and were compensated for their involvement in the study. Additionally, participants with FXS underwent acclimatization training to familiarize them with the actual scanner and environment prior to their PET-MR session to help alleviate their anxiety.

### Data acquisition

Study subjects were scanned for up to 90-min on an integrated whole-body PET-MR (Siemens Biograph mMR) at the MGH Martinos Center for Biomedical Imaging, following an intravenous bolus injection of 5 mCi of 3-[18F]fluoro-5-(2-pyridinylethynyl)benzonitrile ([18F]FPEB) (injected dose: mean 5.15 mCi, SD 0.28; average specific activity at time of injection: 2046 mCi /µmol). Using the published method^48^, the radioligand was locally synthesized at the Gordon Center for Medical Imaging in Massachusetts General Hospital under the guidelines of the Radioactive Drug Research Committee and the approval from the FDA.

Each dynamic study was divided into three scanning sessions of up to 35-min, with breaks between sessions. A structural MRI scan was acquired for each participant at the beginning of each session using a 3-D T1 weighted multi-echo MPRAGE sequence^49^ with the following parameters: TR = 2530 ms, TEs = 1.69, 3.55, 5.41, 7.27 ms, inversion time = 1100 ms, matrix size = 256 × 256 × 176 and voxel size = 1 × 1 × 1 mm^3^. An attenuation map for PET was generated based on the structural MRI data using a hybrid segmentation and atlas-based technique^50^.

### Data processing and analysis

Dynamic list-mode PET data for each scanning session were binned into temporal frames of up to 30 s and were reconstructed and corrected for motion using the following multi-step procedure. First, an initial dynamic reconstruction was performed without correction of attenuation, followed by application of spatial Gaussian smoothing (6-mm full width at half maximum—FWHM) to each reconstructed frame and rigid-body alignment of the activity volume for each frame to a selected reference frame. The attenuation map was then registered to the resulting time-averaged volume and transformed using the previously calculated registration parameters, yielding an attenuation map for each frame. Second, another dynamic reconstruction was performed, incorporating the frame-dependent attenuation map obtained in the first step as well as standard corrections for dead-times, random and scattered coincidences. Note that the attenuation map used during reconstruction also accounted for “static” attenuating medium, such as the scanner’s table and MR head coil. Third, activity volumes in each scanning session were smoothed with a 4-mm FWHM Gaussian filter and rigidly registered to a selected reference frame, followed by another registration to the resulting time-averaged volume. Lastly, all the frames in each session were aligned to a common reference frame, taken as the first session’s time-average image volume. All PET reconstructions were performed using OP-OSEM 3D with 3 iterations and 21 subsets on a 344 × 344 × 127 array with voxel size 2.08 × 2.08 × 2.03 mm^3^. Image registrations were performed using FSL’s flirt method with normalized mutual information as the data consistency criterion and 6 degrees of freedom^51^. To further mitigate motion effects, realignment transformations estimated during the third step were inspected to discard frames associated with substantial frame-to-frame shifts (> 2.5 mm) from the analysis. Linear interpolation was used to compute time points corresponding to discarded frames as well as missing time points between scanning sessions.

Afterwards, the structural MRI scan for each subject was rigidly aligned to PET space (FSL, flirt), followed by non-rigid registration (FSL, fnirt) of the Montreal Neurological Institute (MNI) template to MRI space for definition of regions of interest (ROIs). In keeping with the range of behavioral symptoms associated with FXS, we explored 18 brain regions that have been implicated across neuroimaging studies of FXS^52,53^. [^18^F]FPEB concentration histories were extracted in the following bilateral regions: caudate nucleus, superior temporal gyrus, amygdala, insula, thalamus, anterior cingulate gyrus, primary motor cortex, olfactory cortex, hippocampus, precuneus, prefrontal cortex, pars opercularis, parahippocampal gyrus, pars triangularis, supplementary motor area, superior parietal gyrus, inferior temporal gyrus and cerebellar white matter. Regional time-activity curves (TACs) were fitted using MRTM2^54^ to estimate [^18^F]FPEB binding potential (BP_ND_) with the cerebellum white matter as reference^16^. The t* value was fixed at ${t}^{*}=30 \; \text{min}$^10^ and the ${k}_{2}^{\prime}$ value was estimated by first-pass MRTM analysis of the average TACs^9^. In addition, for visualization purposes, images of [^18^F]FPEB BP_ND_ were generated for each subject by application of MRTM2 analysis at the voxel level. BP_ND_ images were then transformed to MNI space, followed by spatial Gaussian smoothing (5-mm FWHM) and averaging over subjects in each group.

**Figure 3:**
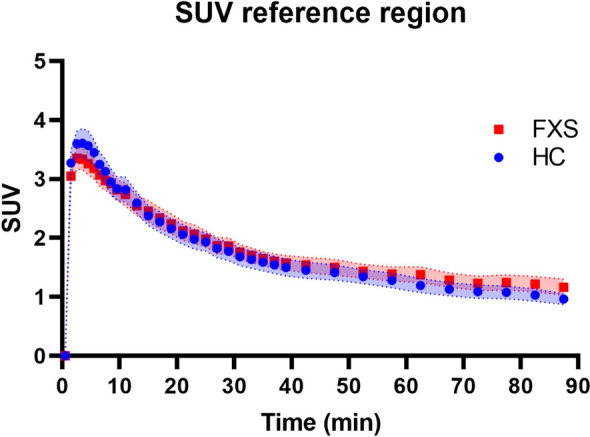
Mean time activity curves in the cerebellum white matter (reference region) for the control subjects (blue circle) and subjects with FXS (red square). Envelope represents standard error of the mean. SUV, standardized uptake value.

Note that no region completely devoid of mGlu5 receptors exists in the human brain^55^. As such, kinetic approaches based on reference region modeling could be biased by individual variations in specific radiotracer uptake. A close examination revealed no substantial differences in reference region TACs which appeared similar for the two groups (Fig. 3).

### Statistical analyses

Unpaired t-tests (2-tailed) were applied to test the null hypothesis of no difference in [^18^F]FPEB BP_ND_ between healthy controls and subjects with FXS for all surveyed regions. The *p*-values were corrected for multiple comparisons by applying the Benjamini, Krieger and Yekutieli false discovery rate method^18^ with *q* = 0.05.

## Data Availability

All datasets generated and/or analyzed during the current study are available from the corresponding authors on reasonable request.
